# Explanations for the Cloudy Evidence That Theory Benefits Health Promotion

**DOI:** 10.3389/fpsyg.2022.910041

**Published:** 2022-07-01

**Authors:** Kevin M. Cummins

**Affiliations:** Department of Public Health, California State University, Fullerton, CA, United States

**Keywords:** health promotion, health psychology, health behavior, theory, scientific representation

## Abstract

Persuasive arguments for using theory have been influential in health behavior and health promotion research. The use of theory is expected to improve intervention outcomes and facilitate scientific advancement. However, current empirical evaluations of the benefits of theory have not consistently demonstrated strong effects. A lack of resolution on this matter can be attributed to several features of the current body of evidence. First, the use of theory may be confounded with other features that impact health-related outcomes. Second, measurement of theory use has not been reliable. Third, the field conflates models and theories. Lastly, the evidentiary status and applicability of theories are not considered. Addressing these challenges during the execution of meta-analyses and designing original research specifically to estimate the benefits of theory could improve research and practice.

## Introduction

The development and application of theory holds a prominent role in health promotion in the USA. It has been suggested that the most efficient and effective health behavior intervention efforts will be facilitated by theory utilization (Noar et al., 2007; Lippke and Ziegelmann, 2008; Michie et al., 2008; Albada et al., 2009; Glanz and Bishop, 2010; Craig et al., 2013; Datta and Petticrew, 2013; Davis et al., 2015). This justifies valuing work that includes an exposition of a project's theoretical basis. It has even been stated that leveraging theory is a necessary criterion for evidence-informed health promotion (DiClemente et al., 2009; Bartholomew et al., 2016).

One expressed motivation for using theory is to access a menu of target constructs for measurement and intervention (Nigg et al., 2002; Noar and Zimmerman, 2005; Crosby et al., 2009; Michie and Prestwich, 2010; Michie et al., 2014; Glanz et al., 2015). In this way, theories are distilled to a list of determinants for a health behavior, many of which have a specified position in a putative causal pathway of a behavioral outcome. These constructs are treated as *hypothesized* mediators of health behaviors (Michie and Prestwich, 2010). It is argued that the use of theory-based constructs was beneficial because the constructs are an index in an organized system for intervention design (Michie and Prestwich, 2010). For example, matrices of intervention components have been mapped to specific theory-based constructs (Michie et al., 2008; Kok et al., 2016). Thus, theory facilitates understanding of related behaviors and provides targets and strategies for interventionists (Glanz, 2005; Michie et al., 2008; Bartholomew et al., 2016).

However, the evidence for superior outcomes from intervention investigations grounded in theory has been mixed. Based on a body of literature reviews published in the first decade of the 2000's, it was concluded that the application of theory was beneficial to the field of health promotion (Glanz and Bishop, 2010). A meta-analysis of web-based behavioral interventions that gauged the types and level of theory utilization found relatively modest effects that were largest for interventions that had the greatest number of reported theory-consistent features (Webb et al., 2010). An analysis of worksite physical activity interventions reported modest improvements in effectiveness when theories were utilized (Taylor et al., 2012). Newer syntheses have been more equivocal (Michielsen et al., 2012; Michie et al., 2014; Prestwich et al., 2014). One recent meta-analysis of physical activity interventions failed to find support for theory-informed approaches (Lock et al., 2020). Another found some evidence that studies invoking theory had notable increases in the effect and consistency of interventions for physical activity (McEwan et al., 2019). However, a review of reviews on physical activity interventions concluded that the effectiveness of theory-based interventions is no greater than those without a stated theoretical basis (Rhodes et al., 2017). The body of work evaluating the benefit of theory has not been convincing enough to end debate over the importance of theory in health promotion (Hagger and Weed, 2019).

### Objective

**Figure 1:**
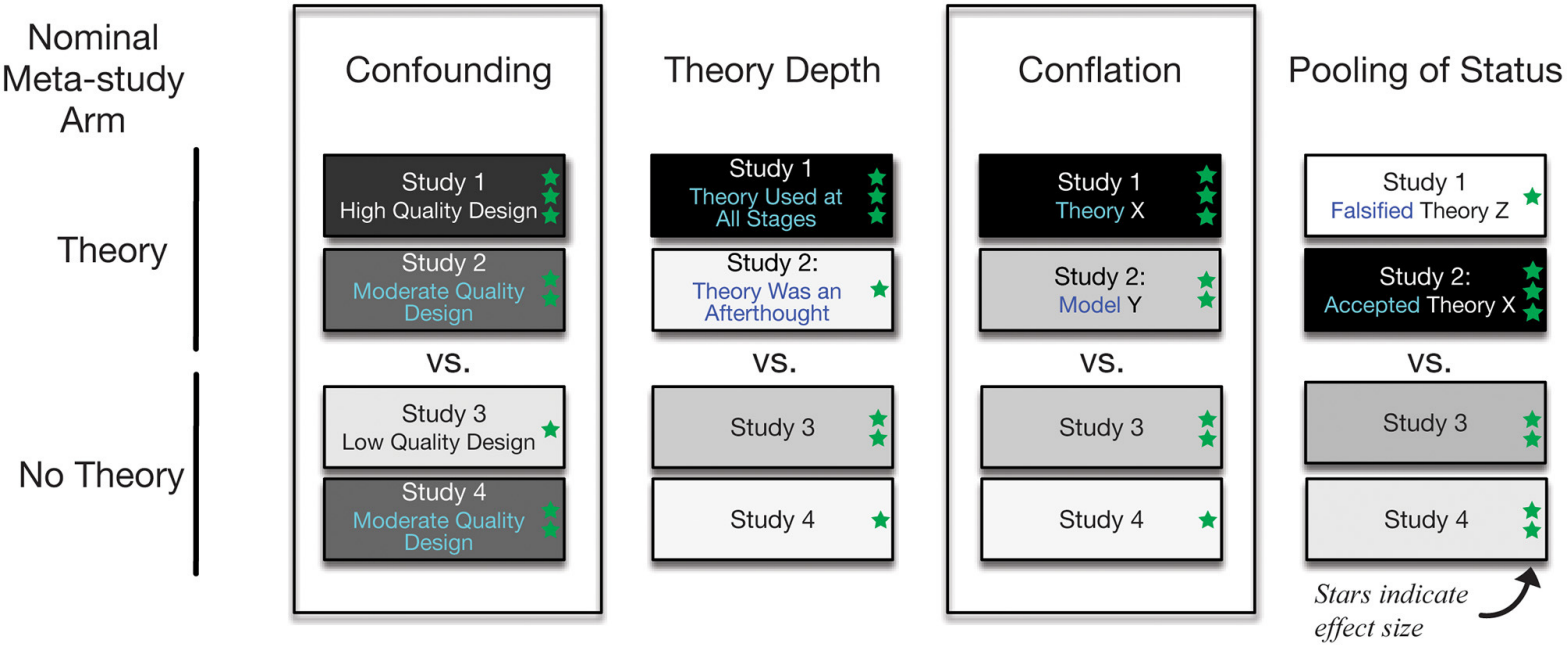
Diagrammatic representations of potential explanations for weak estimated effects attributed to theory in meta-analyses. The diagram provides a visualization of four hypothetical scenarios where a comparison is made between a set of theory-based studies vs. a set that did not use theory. Each box represents one study. The number of green stars and the darkness of the fill represents stronger estimated treatment effects. In each hypothetical scenario, the overall effect in the theory study arm may be attenuated by either confounding, imprecise measurement, conflation, or status pooling. In the example confounding scenario, studies that use theory also have a higher quality experimental design; the effects in Study 3 may be underestimated as an artifact of poor design features. The theory depth scenario exemplifies the situation where theory is invoked in a study, but it was not used in the intervention design, so studies that used theory throughout the intervention development are combined with those that only invoke theory in the research report. In the conflation scenario, studies based on models are treated as being theory-based, so theories are pooled with other types of scientific representations. In the pooling of status scenario, studies using theories that are poor fits to the context or have been demonstrated to be inferior for the particular application and proposed theories are pooled with accepted theories that have been successful and severely tested in the domain of application.

The aim of this paper is to outline several possible explanations for the absence of a conclusive body of work that supports the use of theory in health promotion. It is hoped that compiling and elaborating on these explanations will motivate researchers to consider and address these potential sources of error when evaluating and conducting investigations on theory. The explanations can be categorized into four groups: challenges related to study design, poor discrimination among levels of theory utilization, conflation of models and theory, and pooling of theory status (Figure 1). In the context of the current evidence base, the last three can be considered contributors to measurement error in the assessment of the use of theory.

## Study Designs

Current evaluations primarily rely on comparing patterns *across* studies. Health promotion's core body of original research is not designed to evaluate the use of theory. Although there are some notable exceptions (e.g., Reback et al., 2015), most of the design and analyses presented in the original research reports were not specifically constructed to evaluate the effects of using theory. Consequently, the original research does not include theory as one of the experimental factors. Meta-analyses of theory's effects tend to rely on effect estimates obtained through the synthesis of individual studies contributing estimates of the overall effect of the treatment to the meta-analysis, rather than individual studies contributing within-study estimates of the unique contribution of theory. Because the core of the evidence relied upon is the comparison of theory effects in meta-analyses of *functionally* single-arm studies (i.e., only theory-based or non-theory-based intervention treatments were employed), there is a potential for substantial confounding to influence findings on this topic.

Key confounders of concern are the quality of the overall study design, the intervention features that are not addressed by theory, and the use of prior knowledge in designing interventions. Among reviewed studies of cancer screening, all studies failing to invoke theory in the research report scored the lowest on a structured study quality scale (Noar et al., 2007); although, there are notable counterexamples where study quality is not strongly related to theory (Albada et al., 2009). There is also potential confounding between projects that invoke theory and the type of care taken to construct and execute an intervention (Glanz and Bishop, 2010). Assessment of intervention effects on health behavior is further clouded because no statistical adjustment is made for differences in the potency of interventions to modify directly targeted constructs. For some health behaviors, the success of interventions is related to dosage (e.g., Haller et al., 2016). Further, the same content delivered via different modalities or interventionists can affect potency (Project MATCH Research Group, 1998; Meier et al., 2005; Carey et al., 2007; Cadigan et al., 2015). It has been understood that inclusion of targeted and proximal mediators in the measurement regime of an intervention study can assist in gauging the potency and play a role in explaining the success, or failure, of an intervention (Nigg et al., 2002; Rothman, 2011); thus, there has been recognition that potency is important, but this has not yet been adequately addressed during reviews of the effect of theory. The use of theory does not immunize an intervention study from weak execution or poor design, so comparisons across studies present analysis and interpretation challenges. Further, the differences in design could attenuate or accentuate the outcome comparisons between theory and non-theory anchored interventions. This could happen through the increase in effect estimation bias resulting from confounding or a reduction in the signal-to-noise ratio by not addressing the variance attributable to design features such as dosage.

The most important confounder may be difficult to disentangle under the field's current approach to its treatment of theory. Because of the emphasis on using theory, the field tends to record its knowledge in theories and models; there may be few opportunities to cleanly compare an intervention informed by prior research without its evidence-base coinciding with knowledge represented in published theories. Newly identified determinants may be added to a theory. A notable example is the addition of perceived behavioral control to the Theory of Reasoned Action (Ajzen, 2011). Another example is the Health Belief Model. The supplementation of a self-efficacy construct to the original formulation of the Health Belief Model was in recognition of the importance of this construct as an important determinant of health behavior (Rosenstock et al., 1988; Norman and Brain, 2005). It may be difficult to distinguish between interventions and studies that are truly informed by theory and those informed by a thorough literature search of empirically identified risk and protective factors. Because of this, the field should give the greatest deference to evidence based on original research specifically designed to address the question of theory's benefit.

## Distinguishing Between Invoking Theory and Using Theory

Even among reviews reporting at least one supportive association between theory usage and outcomes, effect sizes have been generally small, inconsistent, only marginally significant, or questionable upon inspection of the interval estimates (Kim et al., 1997; Ammerman et al., 2002; Noar et al., 2007; Taylor et al., 2012). This has left us in a situation where we are obliged to be cautious in predicting that health promotion theory, in its current form, will substantially raise the tide of intervention effectiveness. It is possible that a lack of large effect sizes could be a result of the attenuation caused by poor measurement of theory use and weakness in how experimental comparisons are constructed. However, in one meta-analysis where the manner in which theory was employed in the study was evaluated, the effects were weak at best (Prestwich et al., 2014).

In much of the literature, analyses of the benefits of theory often rely on markers of theory use that may be weakly valid for measuring the characteristics that proponents of theory use envision. Theory use is often dichotomously scored based on whether or not a theory was invoked in a published report (Kim et al., 1997; Ammerman et al., 2002; Noar et al., 2007; Albada et al., 2009; Gardner et al., 2011). There are reports of researchers admitting to only creating a veneer of theory incorporation just to please reviewers (Goodson, 2010). Thus, there may be no difference in intervention techniques among some projects invoking theory and those that do not (Michie et al., 2009). Yet, those that only invoke theory have been categorized as being theory-based in some meta-analyses. This is in part a consequence of how research has been reported. Many commentators have recognized that research reports rarely include an adequate description of the nexus between theory and choices made in the engineering of the intervention studies (Albada et al., 2009; Michie et al., 2009; Michie and Prestwich, 2010; Gardner et al., 2011; Hagger and Weed, 2019). Even if adequate detail is provided, the estimated relationship between theory use and intervention effects is attenuated by the dichotomization in the measurement of theory use (MacCallum et al., 2002).

Several authors have presented means to ameliorate measurement error with the use of structured instruments for assessing the use of theory (Michie and Prestwich, 2010) and recommendations for the use of structured descriptions of the way in which theory influenced a given study (Bartholomew et al., 2016; Peskin et al., 2017; Pot et al., 2018). Research questions, study design, intervention engineering, results interpretation, and conceptual integration can each be driven by theory. The key point is that when research that uses theory only as a veneer in a research report is grouped with investigations that are more richly anchored in theory, evidence in support of theory can be diluted by weak effects found in the studies that superficially used theory.

## Conflation of Models and Theory

Much of the theory-related vocabulary in health promotion is ambiguous (Bartholomew and Mullen, 2011). There are several health promotion-related expositions about theory that provide definitions of scientific theory (Glanz and Bishop, 2010; Goodson, 2010; Glanz et al., 2015; Nilsen, 2015). The definitions vary (Table 1) but can be partially reconciled; however, the broadly divergent *application* of the terminology is often inconsistent with the definitions. This is highlighted by the common application of theory as a set of constructs to measure and target in intervention research. None of the definitions identify theory as lists of determinants (Table 1). Other ways in which the explicit definitions differ from terminological usage are using theory as an umbrella term for all types of scientific representations (e.g., models, theory), a term used for causal explanations (Datta and Petticrew, 2013; Hawe, 2015), or as the articulated rationale of an intervention (Davidoff et al., 2015). Frequently, the discussion of theory does not clearly distinguish which recommendations apply equally to scientific representations titled model and those titled theory (Glanz and Bishop, 2010).

**Table 1:** Exemplar definitions of theory and models found in the health behavior literature.

**Scientific representation type**	**Glanz et al. (2015)^**†**^**	**Simons-Morton and Lodyga (2021)^**†**^**	**Goodson (2010)^**†**^**	**Coreil (2010)**
Theory	A set of interrelated concepts, definitions, and propositions that present a systematic view of events or situations by specifying relations among variables in order to explain and predict events or situations (p. 26).	The possible relationships between constructs or sometimes a hypothesis supported by data. A theory is not necessarily the truth, but it describes relationships, defines terms, and is stated in such a way as to be testable (p. 25).	The end result, the outcome, the outgrowth of a dynamic process of asking and answering very specific types of questions (those concerned with causes, or “why”) (p. 225).	A set of interrelated concepts, constructs, and propositions that present a systematic view of a domain of study for the purpose of explaining and predicting a phenomenon (p. 69).
Model	No explicit definition. Models draw on a number of theories to help understand a specific problem in a particular setting or context (p. 28).	It was not addressed.	A representation of a given phenomenon, or reality (p. 225).	A heuristic device for organizing components of a domain of phenomena to show relationships between the parts and the outcome of interest (p. 26).

*^†^The authors also provide expositions that discuss characteristics of theories and alternative definitions presented by others*.

Fidelity to a taxonomy that differentiates these types of scientific representations will aid evaluation and provide clarity to recommendations related to different types of scientific representations; however, a consistent taxonomy has yet to be adopted and applied in practice by the field. The conflation of models and theory may help explain the current state of empirical evidence regarding the benefits of theory. When weak models are pooled with strong theory, we should expect estimates of the effect of theory to be attenuated. As in other fields, we should expect established theory to provide strong and consistent tools for intervention development, whereas the value of models will be highly dependent on the match between the model, the specific situation, and the modeling goals because models require trade-offs among generality, precision, and realism (Levins, 1966).

## A Common Link Between The Use Of Models and Theory

The prevalent way the term theory is used in health promotion is described by Glanz et al. (2015). Citing Bandura, the function of theory is described by Glanz et al. as cataloging determinants of health behaviors (Bandura, 1986; Glanz et al., 2015). As mentioned above, the most common use of models and theory in interventions and study design is via targeting constructs found in a named theory or model. However, the constructs that are utilized in a specific study typically represent only a subset of a theory's constructs and are sourced from multiple theories (Noar et al., 2007; Albada et al., 2009; Michie et al., 2009; Prestwich et al., 2014). Notably, some original reports fail to include references to any particular theory even though constructs found in theories were included in the research. In a review of print communication interventions, 96% of studies included concepts that authors traced to a referenced theory, although not always to the correct theory (Noar et al., 2007). Of the complement group of studies, 60% used a concept that qualified as a theoretical concept even though an associated theory was not referenced. In Noar's review, the mean number of concepts was four, which indicates that most studies use fewer constructs than in the referenced theories. In another review, only 9% of studies were found to have used all the constructs of a theory in an intervention (Prestwich et al., 2014).

The distinction between models and theory should not matter where theory and models are simply used as an unstructured list of constructs. Models and theory both can function as construct lists. Where this has been the main function of theory, the conflation between models and theories is of little consequence in practice. Further, suppose construct selection is motivated by rationale disconnected from features of the theory other than its constructs list. In that case, intervention effects may not merit attribution to a specific theory, or theory in general. This is particularly salient if the construct(s) are not unique to or dependent on a theory. For example, self-efficacy is found in many named theories and models. The use of self-efficacy, on its own, provides little evidence in support of a particular theory. Finally, suppose the completeness of the construct set in a theory is important. In that case, the unadjusted pooling of results from studies using complete sets with studies using incomplete sets of constructs would be expected to attenuate the estimated effects of theory. Most meta-analyses have not addressed the completeness nor the rationale for construct selection.

## Misclassification of Theory Status

Another consideration is the paucity of evaluation and discussion of the *specific* theory used in studies. Allowing any theory or model to suffice means suboptimal models will dilute the effects of stronger models when their individual effects are pooled in meta-analyses. This is underappreciated. There is a recognition that some theories may be inappropriate for the contexts in which they were applied (Michie et al., 2009; Gardner et al., 2011), but the verisimilitude of candidate theories have not been adequately taken into consideration [see Popper (1994) and Brink (2017) for a discussion of the verisimilitude of theories]. More plainly, if studies using theories that are poor or are not applicable are categorized as being theory-based then the effect estimates for using applicable theory would be expected to be attenuated. Researchers evaluating theory's benefit should consider discriminating between theories utilized within their domains of application and those used outside their domains. Research in this area would greatly benefit from posturing toward determining if applicable theory is beneficial, rather than if any theory is beneficial.

The evidentiary status of the theory should also be considered; the absence of well-established theory for particular health behaviors does not negate this issue. Rather, this would support misclassification as a potential explanation for the absence of a coherent, strong, and consistent evidence base for the benefits of the use of theory. It seems untenable to suggest that any theory of any evidentiary status or development stage should suffice or be considered equivalent in a meta-analysis to an accepted theory because one would only need to declare their rationale in the form of a model (or theory) to meet the criteria for having used theory. After a theory has been evaluated and subsequently become regarded as at least approximately correct for a particular context, this theory should be used in practice. Unfortunately, one of the consistent challenges in applying theory is choosing which theory to apply (Goodson, 2010; Glanz et al., 2015). There are often no theories that are clearly the appropriate choice for application; at best, there are alternatives. This is a strong indicator that the field does not have theories that are generally considered correct; where the field has accepted a certain theory for a particular context or domain, then future reviews should classify theory use based on whether or not accepted theory has been used or not. This contrasts with the common approach of comparing any theory to no theory.

In summary, when studies evaluate the benefits of using theory, the most important feature that can be recorded is if the health behavior is consistent with the theory's domain of application and the status of the theory (e.g., proposed, accepted). Currently, researchers in health promotion rarely distinguish among the evidentiary status of different theories and in meta-analytic reviews implicitly classify all as if they were accepted theories for the health behaviors under study. The estimated benefits of using theory will likely be diluted in evaluations that mix theories and varied evidentiary status or if inapplicable theory is used.

## Discussion

Several challenges to evaluating the benefit of theory in health promotion have been presented (Table 2). The easiest to address is confounding. Confounding can be mitigated with improved study design. The best approach is to focus on original research that compares interventions based on theory against interventions not based on theory in the same research protocol. Meta-analyses could then be limited to original research designed to evaluate theory. Within-study estimates of effect should at least be included in the sensitivity analyses of meta-analyses.

**Table 2:** Challenges and mitigations in the evaluation of theory.

**Scenario**	**Confounding**	**Theory depth**	**Conflation**	**Status pooling**
Synopsis	Theory-based studies have better design and implementation features than non-theory-based studies.	Theory was used to different degrees in studies designated as theory-based.	Models are treated equally to theories.	Theories with different evidentiary statuses are pooled.
Mitigations	Utilize original research designed to compare theory-based interventions with evidence-informed atheoretical interventions. Conduct sensitivity analysis during meta-analysis to assess, adjust for and moderate on study quality.	Report specific features of each study that were determined by theory application. Limit and adjust for type and level of theory application.	Apply lexical and conceptual distinctions between models and theory. Include analyses that limit evaluations to theory.	Differentiate between untested theories that have merely been proposed from those that have survived strong tests and are considered correct in application.

A related area to consider is the recognition that not all theories and models should be equally valuable for every application. Increasingly, evaluations of the benefit of theory have included comparisons between different theories and models (McEwan et al., 2019; Sanaei Nasab et al., 2019). This will be useful for addressing simple questions about theory and provide avenues to address numerous challenges that arise when attempting to take an experimental medicine approach to health behaviors and health promotion (Sheeran et al., 2017; Rothman and Sheeran, 2020). This can be particularly valuable where protocols address multiple causal pathways. The findings can help form an empirically anchored knowledge base that is available to guide research and practice irrespective of theory-related findings.

One of the reasons the experimental medicine approach may provide valuable service to public health is the categorical blurring among different types of scientific representations and their status. There is frequently a lack of *functional* distinction between models and theory (Bartholomew and Mullen, 2011). This can be attributed to the loose conceptual and lexical treatment of theory in health promotion research and practice. Increasing the field's lexical and conceptual precision will enable better communications and assessment of theory.

Health behavior researchers should not discount theory's potential to provide guidance for research and practice. However, health promotion workers should recognize that the evidence base is stacked against finding clean evidence to support the use of theory as it is currently construed. The current dominance of indirect evidence to evaluate theory's benefit, combined with the indeterminate usage of theory in individual studies and pooling of theories, results in several deficiencies in the evidence base. It is possible that by recognizing the difference between models and theory and the evidentiary status of a theory, substantial variability in the service provided by different theories and models will be uncovered. Until we have better resolution, caution should be incorporated into recommendations related to theory in health promotion.

## Author Contributions

KC conceived the concept and organization of the paper and contributed to the interpretation, development, and finalization of the manuscript.

## Funding

Research support from the School of Social Work at San Diego State University through the Joint Doctoral Program in Interdisciplinary Research on Substance Use was critical to the original development of this work. Completion was made possible through research support from California State University, Fullerton.

## Conflict of Interest

The author declares that the research was conducted in the absence of any commercial or financial relationships that could be construed as a potential conflict of interest.

## Publisher's Note

All claims expressed in this article are solely those of the authors and do not necessarily represent those of their affiliated organizations, or those of the publisher, the editors and the reviewers. Any product that may be evaluated in this article, or claim that may be made by its manufacturer, is not guaranteed or endorsed by the publisher.

## References

[B1] Ajzen I.. (2011). “The theory of planned behavior,” in Handbook of Theories of Social Psychology, eds P. A. van Lange, A. W. Kruglanski, and E. T. Higgins (New York, NY: Lawrence Erlbaum Associates).

[B2] Albada A. Ausems M. Bensing J. M. van Dulmen S. (2009). Tailored information about cancer risk and screening: a systematic review. Patient Educ. Couns. 77, 155–171. 10.1016/j.pec.2009.03.005 19376676

[B3] Ammerman A. S. Lindquist C. H. Lohr K. N. Hersey J. (2002). The efficacy of behavioral interventions to modify dietary fat and fruit and vegetable intake: a review of the evidence. Prev. Med. 35, 25–41. 10.1006/pmed.2002.1028 12079438

[B4] Bandura A.. (1986). Social Foundations of Thought and Action: a Social Cognitive Theory. Englewood Cliffs, NJ: Prentice-Hall.

[B5] Bartholomew L. K. Markham C. M. Ruiter R. A. C. Fernández M. E. Kok G. Parcel G. S. (2016). Planning Health Promotion Programs: An Intervention Mapping Approach. San Fransisco, CA: John Wiley and Sons.

[B6] Bartholomew L. K. Mullen P. D. (2011). Five roles for using theory and evidence in the design and testing of behavior change interventions. J. Public Health Dent. 71, S20–S33. 10.1111/j.1752-7325.2011.00223.x 21656946

[B7] Brink C.. (2017). “Verisimilitude,” in A Companion to the Philosophy of Science, eds Newton-Smith (New York: Blackwell Publishing Ltd).

[B8] Cadigan J. M. Haeny A. M. Martens M. P. Weaver C. C. Takamatsu S. K. Arterberry B. J. (2015). Personalized drinking feedback: a meta-analysis of in-person versus computer-delivered interventions. J. Consult. Clin. Psychol. 83, 430. 10.1037/a0038394 25486373 PMC4380651

[B9] Carey K. B. Scott-Sheldon L. A. Carey M. P. DeMartini K. S. (2007). Individual-level interventions to reduce college student drinking: a meta-analytic review. Addict. Behav. 32, 2469–2494. 10.1016/j.addbeh.2007.05.004 17590277 PMC2144910

[B10] Coreil J.. (2010). Social and Behavioral Foundations of Public Health. Thousand Oaks, CA: Sage.

[B11] Craig P. Dieppe P. Macintyre S. Michie S. Nazareth I. Petticrew M. (2013). Developing and evaluating complex interventions: the new medical research council guidance. Int. J. Nurs. Stud. 50, 587–592. 10.1016/j.ijnurstu.2012.09.010 23159157

[B12] Crosby R. A. Kegler M. C. DiClemente R. J. (2009). “Theory in health promotion practice and research.” in Emerging Theories in Health Promotion Practice and Research (San Francisco: Jossey-Bass),3–18.

[B13] Datta J. Petticrew M. (2013). Challenges to evaluating complex interventions: a content analysis of published papers. BMC Public Health 13, 568. 10.1186/1471-2458-13-568 23758638 PMC3699389

[B14] Davidoff F. Dixon-Woods M. Leviton L. Michie S. (2015). Demystifying theory and its use in improvement. BMJ Qual. Saf. 24, 228–238. 10.1136/bmjqs-2014-003627 25616279 PMC4345989

[B15] Davis R. Campbell R. Hildon Z. Hobbs L. Michie S. (2015). Theories of behaviour and behaviour change across the social and behavioural sciences: a scoping review. Health Psychol. Rev. 9, 323–344. 10.1080/17437199.2014.941722 25104107 PMC4566873

[B16] DiClemente R. J. Crosby R. A. Kegler M. C. (2009). Emerging Theories in Health Promotion Practice and Research. San Fransisco, CA: John Wiley and Sons.

[B17] Gardner B. Wardle J. Poston L. Croker H. (2011). Changing diet and physical activity to reduce gestational weight gain: a meta-analysis. Obes. Rev. 12, e602–e620. 10.1111/j.1467-789X.2011.00884.x 21521451

[B18] Glanz K.. (2005). Theory at a Glance: a Guide for Health Promotion Practice. (2nd ed.). Bethesda: U.S. Dept. of Health and Human Services, National Cancer Institute.

[B19] Glanz K. Bishop D. B. (2010). The role of behavioral science theory in development and implementation of public health interventions. Annu. Rev. Public Health 31, 399–418. 10.1146/annurev.publhealth.012809.103604 20070207

[B20] Glanz K. Rimer B. K. Viswanath K. (2015). Health Behavior: Theory, Research, and Practice. (5th ed.) San Fransisco: Jossey-Bass.

[B21] Goodson P.. (2010). Theory in Health Promotion Research and Practice: Thinking Outside the Box. Sudbury, Mass: Jones and Bartlett.

[B22] Hagger M. S. Weed M. (2019). DEBATE: Do interventions based on behavioral theory work in the real world. Int. J. Behav. Nutr. Phys. Act 16, 36. 10.1186/s12966-019-0795-4 31023328 PMC6482531

[B23] Haller M. Norman S. B. Cummins K. Trim R. S. Xu X. Cui R.. (2016). Integrated cognitive behavioral therapy versus cognitive processing therapy for adults with depression, substance use disorder, and trauma. J. Subst. Abuse Treat 62, 38–48. 10.1016/j.jsat.2015.11.005 26718130

[B24] Hawe P.. (2015). Lessons from complex interventions to improve health. Annu. Rev. Public Health 36, 307–323. 10.1146/annurev-publhealth-031912-114421 25581153

[B25] Kim N. Stanton B. Li X. Dickersin K. Galbraith G. (1997). Effectiveness of the 40 adolescent AIDS-risk reduction interventions: a quantitative review. J. Adolesc. Health 20, 204–215. 10.1016/S1054-139X(96)00169-3 9069021

[B26] Kok G. Gottlieb N. H. Peters G.-J. Y. Mullen P. D. Parcel G. S. Ruiter R. A. C.. (2016). A taxonomy of behaviour change methods: an intervention mapping approach. Health Psychol. Rev. 10, 297–312. 10.1080/17437199.2015.1077155 26262912 PMC4975080

[B27] Levins R.. (1966). The strategy of model building in population biology. Am. Sci. 54, 421–431.

[B28] Lippke S. Ziegelmann J. P. (2008). Theory-based health behavior change: developing, testing, and applying theories for evidence-based interventions. Appl. Psychol. 57, 698–716. 10.1111/j.1464-0597.2008.00339.x

[B29] Lock M. Post D. Dollman J. Parfitt G. (2020). Efficacy of theory-informed workplace physical activity interventions: a systematic literature review with meta-analyses. Health Psychol. Rev. 15, 483–507. 10.1080/17437199.2020.1718528 31957559

[B30] MacCallum R. C. Zhang S. Preacher K. J. Rucker D. D. (2002). On the practice of dichotomization of quantitative variables. Psychol. Methods 7, 19. 10.1037/1082-989X.7.1.19 11928888

[B31] McEwan D. Beauchamp M. R. Kouvousis C. Ray C. M. Wyrough A. Rhodes R. E. (2019). Examining the active ingredients of physical activity interventions underpinned by theory versus no stated theory: a meta-analysis. Health Psychol. Rev. 13, 1–17. 10.1080/17437199.2018.1547120 30412685

[B32] Meier P. S. Barrowclough C. Donmall M. C. (2005). The role of the therapeutic alliance in the treatment of substance misuse: a critical review of the literature. Addiction 100, 304–316. 10.1111/j.1360-0443.2004.00935.x 15733244

[B33] Michie S. Jochelson K. Markham W. A. Bridle C. (2009). Low income groups and behaviour change interventions: a review of intervention content, effectiveness and theoretical frameworks. J. Epidemiology Community Health 63, 610–622. 10.1136/jech.2008.078725 19386612

[B34] Michie S. Johnston M. Francis J. Hardeman W. Eccles M. (2008). From theory to intervention: mapping theoretically derived behavioural determinants to behaviour change techniques. Appl. Psychol. 57, 660–680. 10.1111/j.1464-0597.2008.00341.x

[B35] Michie S. Prestwich A. (2010). Are interventions theory-based? development of a theory coding scheme. Health Psychol. 29, 1. 10.1037/a0016939 20063930

[B36] Michie S. F. West R. Campbell R. Brown J. Gainforth H. (2014). ABC of Behaviour Change Theories. Great Britain: Silverback Publishing.

[B37] Michielsen K. Chersich M. Temmerman M. Dooms T. Van Rossem R. (2012). Nothing as Practical as a Good Theory? the theoretical basis of HIV prevention interventions for young people in sub-saharan africa: a systematic review. AIDS Res. Treat 2012, 345327. 10.1155/2012/345327 22900155 PMC3415137

[B38] Nigg C. R. Allegrante J. P. Ory M. (2002). Theory-comparison and multiple-behavior research: common themes advancing health behavior research. Health Educ. Res. 17, 670–679. 10.1093/her/17.5.670 12408211

[B39] Nilsen P.. (2015). Making sense of implementation theories, models and frameworks. Implement. Sci. 10, 53. 10.1186/s13012-015-0242-0 25895742 PMC4406164

[B40] Noar S. M. Benac C. N. Harris M. S. (2007). Does tailoring matter? meta-analytic review of tailored print health behavior change interventions. Psychol. Bull. 133, 673. 10.1037/0033-2909.133.4.673 17592961

[B41] Noar S. M. Zimmerman R. S. (2005). Health behavior theory and cumulative knowledge regarding health behaviors: are we moving in the right direction. Health Educ. Res. 20, 275–290. 10.1093/her/cyg113 15632099

[B42] Norman P. Brain K. (2005). An application of an extended health belief model to the prediction of breast self-examination among women with a family history of breast cancer. Br. J. Health Psychol. 10, 1–16. 10.1348/135910704X24752 15826330

[B43] Peskin M. F. Hernandez B. F. Gabay E. K. Cuccaro P. Li D. H. Ratliff E.. (2017). Using intervention mapping for program design and production of iCHAMPSS: an online decision support system to increase adoption, implementation, and maintenance of evidence-based sexual health programs. Front. Public Health 5, 203. 10.3389/fpubh.2017.00203 28848729 PMC5554483

[B44] Popper K. R.. (1994). The Myth of the Framework: in Defense of Science and Rationality. London: Routledge.

[B45] Pot M. Ruiter R. A. C. Paulussen T. W. G. M. Heuvelink A. de Melker H. E. van Vliet H. J. A.. (2018). Systematically developing a web-based tailored intervention promoting HPV-vaccination acceptability among mothers of invited girls using intervention mapping. Front. Public Health. 6:226. 10.3389/fpubh.2018.00226 30356852 PMC6190841

[B46] Prestwich A. Sniehotta F. F. Whittington C. Dombrowski S. U. Rogers L. Michie S. (2014). Does theory influence the effectiveness of health behavior interventions? meta-analysis. Health Psychol. 33, 465. 10.1037/a0032853 23730717

[B47] Project MATCH and Research Group (1998). Therapist effects in three treatments for alcohol problems. Psychother. Res. 8, 455–474. 10.1093/ptr/8.4.455

[B48] Reback C. J. Fletcher J. B. Shoptaw S. Mansergh G. (2015). Exposure to theory-driven text messages is associated with HIV risk reduction among methamphetamine-using men who have sex with men. AIDS Behav. 19, 130–141. 10.1007/s10461-014-0985-7 25563501

[B49] Rhodes R. E. Janssen I. Bredin S. S. D. Warburton D. E. R. Bauman A. (2017). Physical activity: Health impact, prevalence, correlates and interventions. Psychol. Health 32, 942–975. 10.1080/08870446.2017.1325486 28554222

[B50] Rosenstock I. M. Strecher V. J. Becker M. H. (1988). Social learning theory and the health belief model. Health Educ. Q. 15, 175–183. 10.1177/109019818801500203 3378902

[B51] Rothman A. J.. (2011). Be prepared: capitalizing on opportunities to advance theory and practice. J. Public Health Dent. 7 (Suppl. 1), S49–50. 10.1111/j.1752-7325.2011.00240.x 21656952

[B52] Rothman A. J. Sheeran P. (2020). What is slowing us down? six challenges to accelerating advances in health behavior change. Ann Behav Med 54, 948–959. 10.1093/abm/kaaa090 33416843

[B53] Sanaei Nasab H. Yazdanian M. Mokhayeri Y. Latifi M. Niksadat N. Harooni J.. (2019). The role of psychological theories in oral health interventions: a systematic review and meta-analysis. Int. J. Dent. Hyg. 17, 142–152. 10.1111/idh.12386 30702796

[B54] Sheeran P. Klein W. M. P. Rothman A. J. (2017). Health behavior change: Moving from observation to intervention. Annu. Rev. Psychol. 68, 573–600. 10.1146/annurev-psych-010416-044007 27618942

[B55] Simons-Morton B. Lodyga M. (2021). Behavior Theory in Public Health Practice and Research. Burlington, MA: Jones and Bartlett Learning.

[B56] Taylor N. Conner M. Lawton R. (2012). The impact of theory on the effectiveness of worksite physical activity interventions: a meta-analysis and meta-regression. Health Psychol. Rev. 6, 33–73. 10.1080/17437199.2010.533441

[B57] Webb T. L. Joseph J. Yardley L. Michie S. (2010). Using the internet to promote health behavior change: a systematic review and meta-analysis of the impact of theoretical basis, use of behavior change techniques, and mode of delivery on efficacy. J. Med. Internet Res. 12, e4. 10.2196/jmir.1376 PMC2836773 20164043

